# Mechanical and Dielectric Properties of a Flexible Anisotropic Rubber-Based Composite

**DOI:** 10.3390/nano12132182

**Published:** 2022-06-25

**Authors:** Jie Dong, Chunhai Wang, Xingyu Fan, Liang Wei, Guodong Shen, Runjun Sun, Rong Li

**Affiliations:** 1 School of Textile Science and Engineering, Xi’an Polytechnic University, Xi’an 710048, China; xyfan@xpu.edu.cn (X.F.); liangwei@xpu.edu.cn (L.W.); shenguodong@xpu.edu.cn (G.S.); sunrunjun@xpu.edu.cn (R.S.); 2 Key Laboratory of Functional Textile Material and Product (Xi’an Polytechnic University), Ministry of Education, Xi’an 710048, China; 3 State Key Laboratory of Solidification Processing, Northwestern Polytechnical University, Xi’an 710072, China; wangch@nwpu.edu.cn; 4 Xi’an Research Institute of High Technology, Xi’an 710025, China; xss760829@163.com

**Keywords:** rubber-based conductive composites, chopped glass fiber, graphene, flexibility, dielectric properties

## Abstract

Rubber-based conductive composites are widely used in sensors, wearable electronic devices and electromagnetic fields. In this work, by using the two-roll milling and hot-pressing process, chopped glass fiber (CGF) and graphene (Gr) as additives, and acrtlinitrile-brtadiene rubber (NBR) as the matrix, a series of anisotropic flexible rubber-based composites were prepared. Using this preparation method, both CGF and Gr additives were directly arranged in the material. When the content of CGF was 1 wt.%, the tensile strength in both the T and W directions of the material reached 27 MPa and 28 MPa, respectively. When the content of CGF was fixed at 1 wt.% and Gr was 1.5 wt.% and the elongation at break in both directions reached 328% and 347%. By focusing on the comparison of the dielectric differences in the T and W directions in the X band, it was found that the directional arrangement of the additives led to differences in the dielectric properties.

## 1. Introduction

In recent years, the application of electromagnetic waves in military and civilian fields has gained momentum [1,2,3]. However, everything is a double-edged sword. Whether occurring in a military setting or from the transmission of people’s daily mobile phones signals in the civilian field, from 2G to the current 5G, electromagnetic waves bring both convenience and harm to people’s lives. Therefore, the protection of electromagnetic pollution caused by electromagnetic waves has been taken into consideration. Different from hard resin-based or ceramic-based composite materials [4,5,6], due to the high elasticity of rubber, conductive rubber composite materials are used as sensors, thermistors, wearable electronic devices and electromagnetic material [7,8,9,10,11,12,13]. At present, in order to improve the microwave absorption of rubber-based composites, some conductive particles, such as carbon black (CB) [14,15,16], single-wall carbon nanotubes [17], multi-wall carbon nanotubes [18], barium titanate (BT) [19], M-type hexagonal ferrites [20], Pb (Mg_1/3_Nb_2/3_)_0.65_Ti_0.35_O_3_ powder [21] and carbonyl iron [22,23] are added alone. Alternatively, two kinds of conductive particles are added to the rubber matrix, such as CB and carbon nanotubes [24] and CB and strontium ferrite [25]. Graphene is a potential absorbing agent that can prepare microwave absorbing material due to its high diameter/thickness ratio and excellent electrical conductivity. For microwave absorbing materials, simply improving the dielectric properties of the material cannot achieve a better absorption effect on electromagnetic waves. For ideal absorbing materials, two basic principles need to be considered [26]. Some studies [13,27,28,29] have prepared microwave absorbing composites using graphene alone as the absorbing agent. However, due to its high conductivity and strong conjugation, the materials could not meet the impedance matching and attenuation requirement of ideal absorbing material, so it did not have good absorbing properties. Therefore, some scholars adjusted the absorption performance of the material by other means. For example, Gao Y. et al. [30] used graphene as a filler and thermoplastic polyurethane (TPU) as a matrix to study the influence of material foaming and non-foaming on the dielectric and microwave absorbing properties of the prepared composite materials and found that foaming could help to improve dielectric loss of the material and the flexibility. Hu J.N. et al. [31] developed reduced graphene oxide@Fe_3_O_4_ nanopowder to enhance the microwave absorption properties of silicone rubber composites via a facile thermal decomposition method. 

In addition, in order to improve the mechanical properties of rubber-based materials, some studies have undertaken a series of investigations. For example, the effect of interfacial modification on the mechanical properties of a styrene butadiene rubber (SBR) composite was investigated by grafting three modifiers onto a silica surface [32]. Furthermore, the study of material surface modification on the interfacial bonding properties and fatigue properties of rubber-based composites was carried out [33]. By studying the differences in the mechanical properties of rubber-based and resin-based composites with fibers as reinforcements, it was found that in rubber-based composites, the improved bonding between the layers coupled with the flexibility were perhaps the major reason for the improvement in impact performance [34]. Adding conductive particles to the material could effectively improve the conductivity and resistive creep behavior of the rubber matrix composite [12,35,36,37,38]. Some studies have also investigated the effect of fiber content and resorcinol-formaldehyde-latex (RFL) treatment on the chopped aramid fibers reinforced by acrylonitrile-butadiene rubber, finding that adding the aramid fibers effectively improved the wear resistance and the NBR composites via enhancing the hardness and tear strength. However, the effect of fiber straightness on material properties was not considered [39]. There are also some studies in the literature that use the blade-coating technique to prepare a nacre-like styrene-butadiene rubber/crumpled graphene nanosheet heater (NSGH). The comprehensive superior performance of NSGH was due to the well design of the nacre-like structure [40].

In this present work, a very simple and easy way to achieve directional arrangement of fibrous material was introduced: the two-roll milling method. This method is commonly used for preparing rubber materials, but few researchers have paid attention to the phenomenon of directional arrangement of fillers in rubber due to this method. In this experiment, chopped glass fiber (CGF) and graphene (Gr) were selected as fillers and acrtlinitrile-brtadiene rubber (NBR) as a matrix, and a series of anisotropic flexible rubber matrix composites were prepared using the two-roll milling and hot-pressing method. The mechanical, dielectric properties, and flexibility of the prepared composites were investigated. Due to the shearing force of the roller during the preparation process, both chopped glass fiber and graphene showed directional arrangement. Thus, the directional arrangement of the fillers led to anisotropy in the structure and properties of the material.

## 2. Experimental

### 2.1. Materials

NBR powder with an average particle size of 150 mesh used in this experiment was purchased online, which was produced in Shenzhen, China. The length of the CGF used in this article was between 0~3 mm, and the diameter of the single fiber was about 13 μm. The product was from Hangzhou High-tech Composite Materials Co., Ltd., Hangzhou, China. The Gr used in this experiment was a multi-layer structure with resistivity of 0.8 Ω·m, which was obtained from Shaanxi Coal and Chemical Industry Group Co., Ltd., Xi’an, China.

### 2.2. Sample Preparation

Here, the ultrasonic dispersion and high-speed shearing disperser were introduced to mix fillers and rubber thoroughly. At the beginning, different contents of CGFs were placed in a beaker for ultrasonic dispersion for half an hour, the purpose was to make the CGF uniformly dispersed in the clump. Then, the NBR matrix was added into the pre-beaker and stirred with a high-speed shearing machine to obtain a uniformly dispersed composite material. The mixed composites were formed by temperature controlled two-roll milling equipment to prepare a series of chopped glass fiber reinforced rubber matrix composites (CGF/NBRC). The composites were then molded into sheets and subjected to various tests. After optimizing the results, a specific content of CGF and different contents of Gr were selected to compound the composite materials. The preparation process was basically the same as the previous, expecting that both CGF and Gr were added to the beaker at first. In this paper, the samples without fiber or Gr were stated as 0 wt.%. 

### 2.3. Characteristics

**Figure 1:**
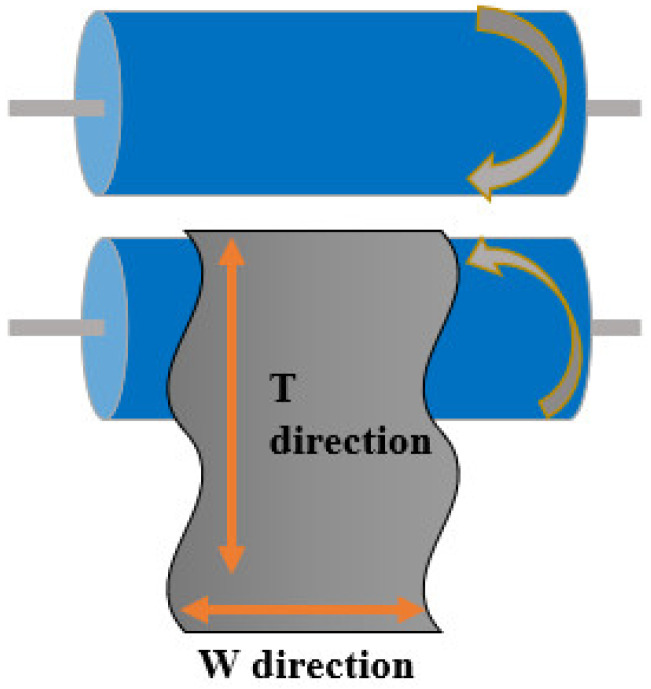
Schematic diagram of the direction determination.

The chemical bond was analyzed by Fourier transform infrared (FT-IR) spectrum (PERKIN ELMER) in the range of 4000–5000cm^−1^. The morphologies and structures of the materials were obtained by scanning electron microscope (SEM FEI Quanta 450). The mechanical properties of tension mode were measured by a universal strength tester according to ISO 37: 2005, and the test shape was a dumbbell shape with a testing size of 20 mm × 5 mm. The dynamic thermomechanical properties of the material were obtained by NETZSCH DMA 242C tester in tension mode with a heating rate of 3 ℃/min from −60 ℃ to 200 ℃ at 10 Hz. The electromagnetic parameters of the material were measured by the network analyzer (Agilent technologies E8362B) using the wave guide method in five bands. The sample sizes in the frequency of 2.6–18 GHz, corresponding to each band, were 72.5 mm × 33.82 mm × 1.0 mm, 47.48 mm × 21.92 mm × 1.0 mm, 34.48 mm × 15.42 mm × 1.0 mm, 22.86 mm × 10.16 mm × 1.0 mm, 15.76 mm × 7.85 mm × 1.0 mm. In this article, we stipulated that the rotation direction of the mill was in the T direction, and the direction perpendicular to the T direction was the W direction, as shown in Figure 1.

## 3. Results and Discussions

### 3.1. FTIR Spectrum of NBR

**Figure 2:**
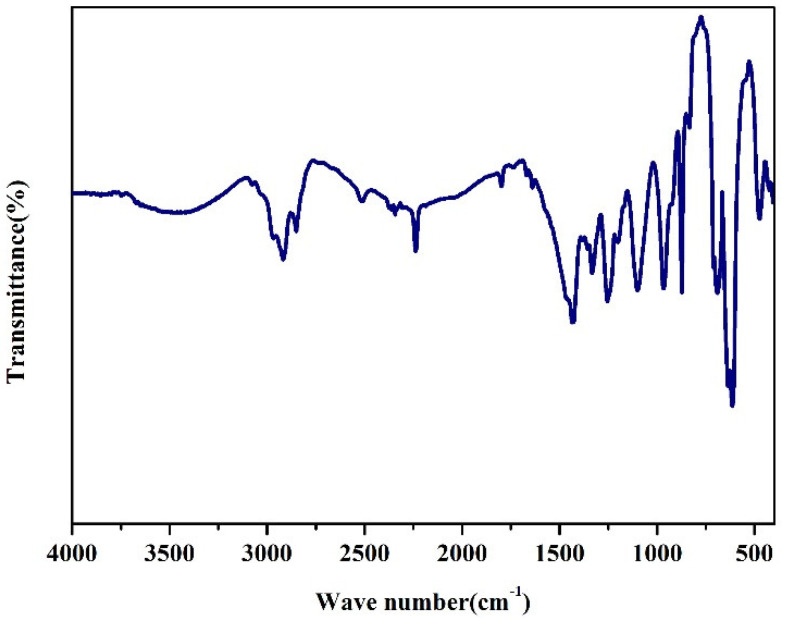
FTIR spectrum of NBR.

The FTIR spectrum was carried out to investigate the structure of NBR, shown in Figure 2. The appearance of the wavenumber at 3478 cm^−1^ proves the existence of O-H bonds in the sample. Then, the wavenumbers corresponding to the stronger peaks are 2930 cm^−1^ and 2850 cm^−1^, respectively, because of the existence of -CH_2_-. The FTIR spectra of the sample reveals that the presence of -CH_2_-deformation vibration structures along the chain as indicated by the presence of the peaks at 1430 cm^−1^. The band at 962 cm^−1^ is indicative of -CH- stretching vibration of R_1_CH = CHR_2_. The peak near 870 cm^−1^ may be attributed to the -CH- stretching vibration of R_1_CH = CH_2_. Due to the existence of spurious peaks, -C ≡ N stretching vibration peaks appear at around 2200 cm^−1^. According to existing reports in the literature, it can be seen that the NBR used in this article is a modified nitrile rubber containing an ester group [41].

### 3.2. Morphologies and Structures

**Figure 3:**
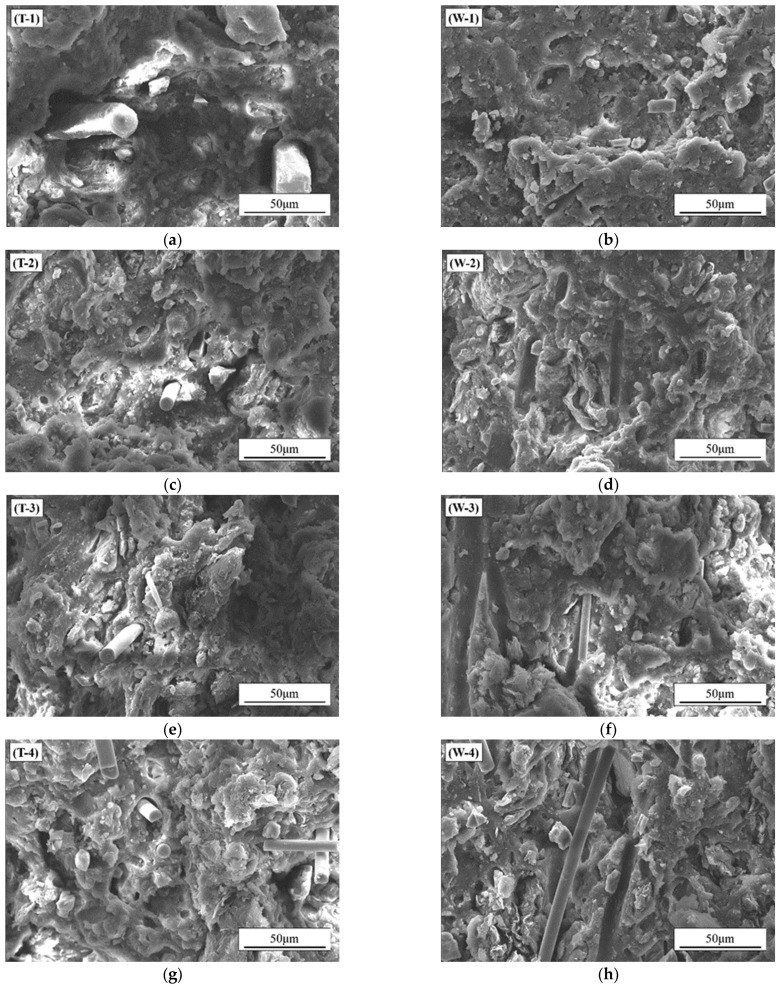

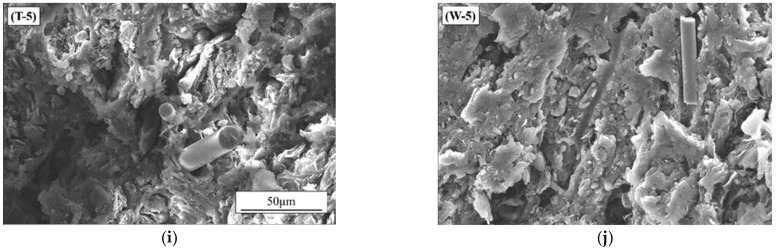
Tensile cross-sectional morphologies of materials with different Gr contents and the same CGF content (1 wt.%). (**a**,**b**): 0 wt.% Gr, (**c**,**d**): 0.5 wt.% Gr, (**e**,**f**): 1.0 wt.% Gr, (**g**,**h**): 1.5 wt.% Gr, (**I**,**j**): 2.0 wt.% Gr.

**Figure 4:**
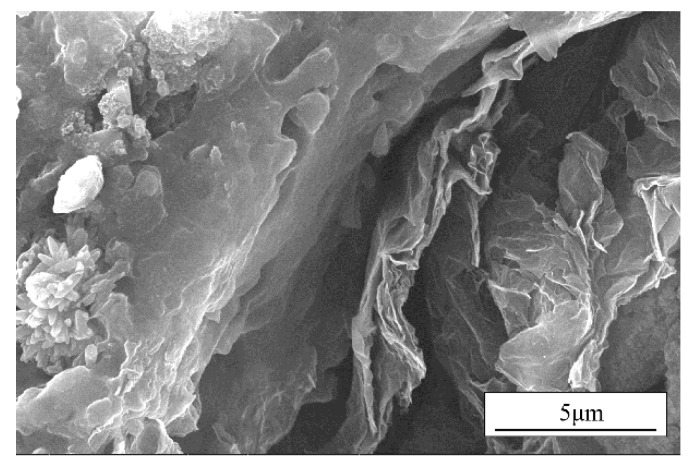
The enlarged imagine of the tensile cross-sectional morphology in the W direction of the material with 0.5 wt.% Gr and 1.0 wt.% CGF.

The prepared samples with CGF content of 1 wt.% and different contents of Gr are measured for tensile properties by a universal strength tester. The tensile properties of the sample materials with CGF content of 1 wt.% and different Gr contents were tested by a universal strength tester. The cross-section of the samples after tensile fracture were observed as shown in Figure 3. As can be seen from Figure 3, the symbol T- in the figure represents the morphology of the samples after tensile strength in the T direction and W- represents the morphology in the W direction. In Figure 3a,c,e,g,i, the direction of the CGF in the material appears to be perpendicular to the cross-sectional direction; that is to say, in line with the stretching direction of the material. This is because in the preparation process, under the shearing force of the rollers, material forms appear in directional arrangement. Additionally, this phenomenon is verified from the cross-sectional view in the W direction. It can be seen that after the material in the W direction is stretched and fractured, some grooves of the CGF are exposed. It can be seen that after the material undergoes tensile fracture in the W direction, part of grooves of the CGF are exposed. Additionally, according to the direction of the grooves, it can also be determined that the distribution direction of the CGF in the material is parallel to the cross-section of the material in the W direction. With the increase of the Gr content, the distribution of Gr in the material gradually increases. The morphology of the sample in the W direction with 0.5 wt.% Gr and 1 wt.% CGF is enlarged, as shown in Figure 4. It can be seen that the distribution of Gr in the material is perpendicular to the fracture section of the material in the W direction. Therefore, the shearing effect of the rollers not only orientate the CGF, but also orient the Gr material. 

### 3.3. Mechanical Properties

#### 3.3.1. Tensile Property of Chopped Glass Fiber Reinforced Rubber Matrix Composites (CGF/NBRC)

**Figure 5:**
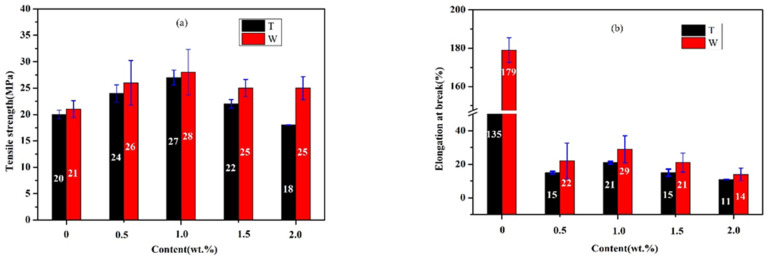
Variation curve of tensile properties of rubber-based composites with different contents of CGF. (**a**) tensile strength, (**b**) elongation at break.

Chopped glass fiber reinforced rubber matrix composites are prepared by adding different contents of CGF into the rubber matrix. The tensile strength and elongation at break of the CGF/NBRC are measured by a universal strength tester. It can be seen from Figure 5 that the anisotropy of the sample due to the directional arrangement leads to the difference in the mechanical properties of the material in the two directions. The tensile strength of the material in the W direction is slightly higher than that in the T direction. Among them, the strength of pure rubber in the T and W directions reached 20 Mpa and 21 Mpa, respectively. Its tensile strength is about 9-times higher than that (2.15 Mpa) of pure NBR prepared by the melt mixing method in the literature [19]. With the increase in CGF, the tensile strength of the material first increases and then decreases. Compared with the pure rubber, in the T direction, when the CGF content is 1 wt.%, the tensile strength of the material is increased by 35%, reaching the maximum value of 27 Mpa. This is because the addition of the fiber parallel to the T direction increases the force of the pure rubber material, and, subsequently, with the increase in CGF content, the tensile properties of the material show a declining trend. Due to the increased brittle CGF, the interaction of original chemical bonds between rubber molecules is weakened. The tensile strength of the material in W direction shows the same trend as the T direction with increasing CGF content. However, from the microstructure of the material, it can be seen that the distribution of CGF in the material is consistent with the T direction. The addition of CGF causes the maximum tensile strength in the W direction to be 2% higher than that in the T direction, and with the increase in CGF content, the deterioration rate of the material is not as obvious as that in the T direction. This is because the tensile deformation of the directional arranged brittle CGF and deformation of the flexible NBR are not synchronized under the action of external force. Figure 5b shows the elongation at break of the rubber composites. It can be seen that the elongation at break of the material also showed anisotropy in the T and W directions, and the elongation at break in the W direction is higher than that in the T direction. The reason for this phenomenon is that under the shearing force of the roller, the chain of the rubber in the T direction has been stretched and arranged neatly. As a result, the elongation at break of the pure rubber material in the W direction is 33% larger than that in the T direction. The addition of brittle substances cut the original neatly arranged molecular chains like scissors. When 1 wt.% CGF is added, the elongation at break in the T and W directions of the materials remains 16% that of pure rubber. Therefore, the addition of CGFs greatly weaken the elastic elongation of the material, while on the other hand, the flexibility of the material is reflected.

**Figure 6:**
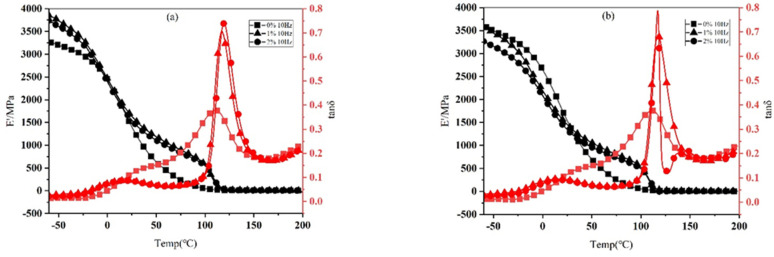
Dynamic thermomechanical properties of the material in tensile mode at a test frequency of 10 Hz with different CGF contents. (**a**) T direction, (**b**) W direction.

The thermodynamic properties of CGF/NBRC under the frequency of 10 Hz in tensile mode is analyzed by DMA. In Figure 6, E’ represents the storage modulus, which reflects the energy stored due to elastic deformation during the deformation process of the material, used to characterize the rigidity of the material. Tanδ is a mechanical loss factor, which represents the ratio of energy dissipated in the form of heat to stored energy, representing the ability of material to lose energy during deformation. When the test frequency is 10 Hz and the addition contents of CGF are 0 wt.%, 1 wt.% and 2 wt.%, the results of the dynamic thermomechanical properties of the CGF/NBRC in tensile mode are shown in Figure 6. It can be seen from Figure 6 that the addition of CGF changes the storge modulus and loss factor of the material. This is because the addition of CGF increases the hardness of the rubber and the movement of the molecular chain is hindered by the CGFs, which reduces the frictional loss within the molecule and increases the storage modulus of the material in the T and W directions; that is, it improves the rigidity of the material. Figure 6a show that the addition of CGF shifts the peak of the tanδ curve of the material to a high temperature, and the peak shape becomes narrower after adding CGFs. Since the addition of CGF restricts the movement of molecules, the resistance that the molecular movement overcomes increases, so the Tg value of the composite material increases and the peak shape narrows macroscopically. As can be seen from Figure 6b, in the W direction, the material exhibits the same trend as in the T direction.

#### 3.3.2. Tensile Property of Graphene and Chopped Glass Fiber Reinforced Rubber Matrix Composites (Gr/CGF/NBRC)

**Figure 7:**
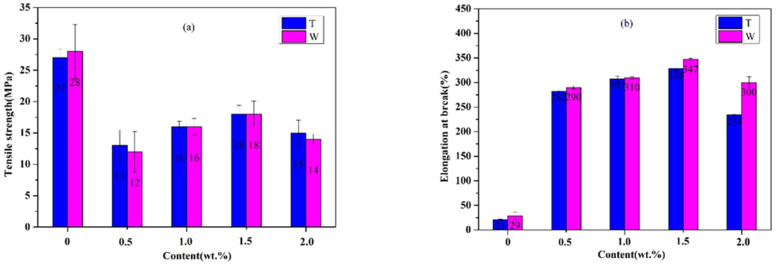
Tensile properties of rubber-based composites with 1 wt.% CGF and different contents of Gr. (**a**) tensile strength, (**b**) elongation at break.

The fixed added amount of CGF is 1 wt.%, and Gr with different contents is added to prepare the graphene/chopped glass fiber reinforced rubber-based composite (Gr/CGF/NBRC). Figure 7a illustrates that with the increase in graphene content, the tensile strength of the composite material increases first and then decreases. When no Gr is added, the tensile strength of the material in the T direction is 27 MPa. As the content of Gr increases to 0.5 wt.%, the tensile strength of the material becomes 13 MPa. When the content increases to 1.5 wt.%, the strength increases to 18 MPa, retaining 67% of the tensile strength (without Gr). It can be seen from Figure 7b that the increase in Gr greatly improves the elongation at break of the material. When no Gr is added, the tensile elongation at break of the material in the T direction is 21%. With the increase in Gr to 0.5 wt.%, the elongation at break increases sharply to 282%. The W direction also shows the same trend as the T direction due to the increase in Gr content. When the content of Gr reaches 1.5 wt.%, the tensile strength and elongation of the material reach the best. Due to the addition of Gr, it acts as a cross-linked agent, making the originally broken bonds cross-link again to form macromolecular chains, thereby increasing the elongation at break of the material.

### 3.4. Dielectric Properties

**Figure 8:**
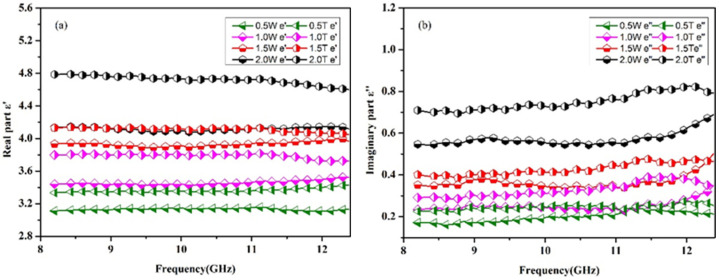
Comparison of complex permittivity of materials with 1wt.% CGF and different Gr contents in T and W directions in the X band. (**a**) real part, (**b**) imaginary part.

**Figure 9:**
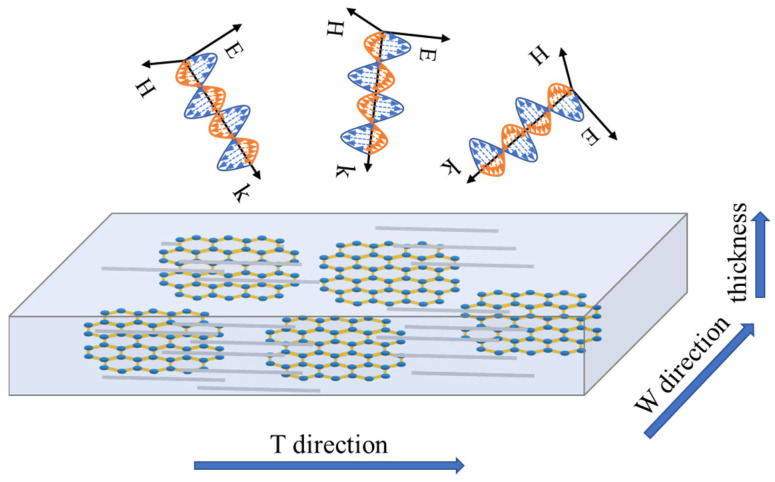
Schematic diagram of material anisotropy.

The materials used in this article are all non-magnetic materials, so u′ = 1 and u″ = 0, in which, u′ and u″ are the real and imaginary parts of complex permeability. The complex permittivity of the composites can be expressed by ε = ε′ − j ε″. The ε′ represents the ability of the material to store charge; that is, the polarization properties of the material. The ε″ represents the ability of the material to covert electromagnetic energy into other forms of energy under the action of the electric field, the energy consumed by the formation of dipoles. The complex permittivity of the Gr/CGF/NBRC in the T direction and W direction are compared in the X band in Figure 8. As can be seen from Figure 8a,b, the material exhibits different real and imaginary parts of the dielectric in the two directions due to anisotropy. At 8.2 GHz, when the addition of Gr is 2 wt.%, the real part of complex permittivity in the T direction is 4.8, while in the W direction it is 4.13. When the addition is 1wt.%, the real part in the T direction is 3.8, while in the W direction it is 3.4. The directional shearing force of the preparation method makes the distribution of absorbent in the material oriented. As a result, the material exhibits different properties in different directions. The distribution state of Gr in the material can also be reflected from the microscopic morphology. The imaginary part of the material also shows the same trend. At 8.2 GHz, when the Gr content is 2 wt.%, the imaginary parts of the material are 0.71 and 0.55 in T and W directions, respectively. Additionally, it can be seen from Figure 8 that with the increase in absorbent content, the difference in the complex permittivity of the composite material in the T and W directions is increased. Due to the high diameter/thickness ratio of Gr, the formation of a conductive network in the thickness direction of the absorber is weaker than that in the diameter direction. Therefore, the dielectric properties of the materials show differences due to anisotropy. A schematic diagram of material anisotropy is shown in Figure 9.

**Figure 10:**
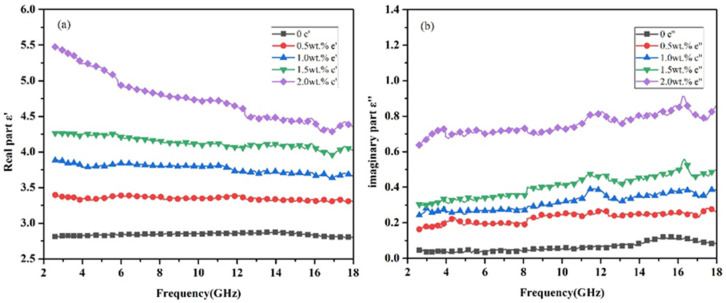
Dielectric properties of rubber-based composites with 1 wt.% CGF and different contents of Gr in the T direction from 2.6 to 18 GHz. (**a**) real part (**b**) imaginary part.

The dielectric properties of the Gr/CGF/NBRC in the T direction in the whole frequency of 2.6–18 GHz are shown in Figure 10. The real part of the complex permittivity of the composite material is given in Figure 10a. It can be seen from Figure 10a that with the increase in Gr, the real part of the composite material shows a gradual increasing trend in the whole band. When the content of Gr increases to 2 wt.%, the real part exhibits the characteristics of dispersion; that is, with the increase in frequency, the real part of the complex permittivity shows a decreasing trend. The real part of complex permittivity of CGF reinforced rubber composite is almost constant at 2.8 over the frequency range from 2.6 to 18 GHz. With the addition of Gr increase, the real part of complex permittivity of the composite material increases steadily. When the Gr content is 2 wt.%, the real part reaches 5.5 at 2.6 GHz. The original network inside the insulating material will be turned on due to the additional increase in conductive Gr. Figure 10b shows the curve of the imaginary part of the dielectric with the increase in Gr content. It can be seen that with the increase in Gr content, the imaginary part of the composite still shows an increasing trend. When the Gr content is 2 wt.%, the imaginary part of the material is about 15-times more than that without Gr. The imaginary part implies the material’s ability to lose electromagnetic waves. The addition of Gr increases the conductive network inside the material. Under the action of the electric field, the movement of electrons in different directions forms dipoles, which, in turn, promotes the occurrence of polarization. For the large diameter/thickness ratio, the increased Gr in the material forms numbers of conductive networks inside the material, thus promoting the occurrence of surface polarization. 

**Figure 11:**
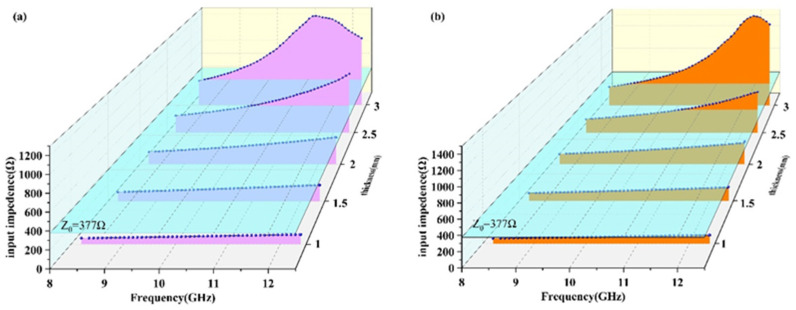
The curves of the input impedance of the material with different thicknesses when the Gr content is 2 wt.%. (**a**) T direction, (**b**) W direction.

As one of the principles of ideal absorbing material, proper impedance matching is beneficial to improve the absorbing performance of the material. Figure 11 shows the input impedance of the material in the T and W direction when the Gr content is 2 wt.% under different thicknesses. The input impedance in free space Z_0_ is 377 Ω. It can be seen from Figure 11 that the impedances of the material in the T direction and W direction are different. Therefore, how to adjust the impedance of the material at a specific thickness is close to or even equal to the impedance of free space will be considered in the next step.

### 3.5. Flexible Properties

**Figure 12:**
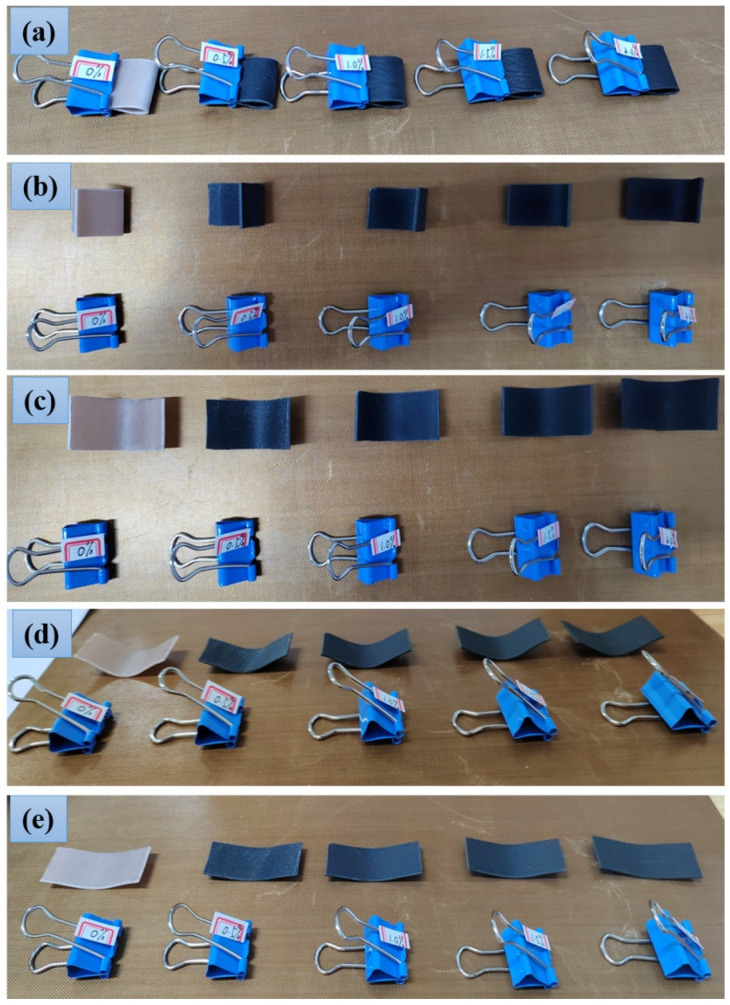
Flexible properties of rubber-based composites. (**a**) initial state, (**b**) the momentary state of releasing the dovetail clip after 30 h, (**c**) releasing the dovetail clip for 10 min, (**d**) releasing the dovetail clip for 30 min, (**e**) releasing the dovetail clip for 1 h.

The flexibility of rubber-based composites containing 1 wt.% CGF and different contents of Gr are characterized, as shown in Figure 12. The sample size is 34.5 mm × 15.4 mm × 1 mm, and the long-side direction is the T direction, which is placed from left to right in line with the increase in Gr content. The samples are aligned along the long side and fixed with dovetail clips, as shown in Figure 12a. After 30 h, the dovetail clips are removed, and the state of the material is shown in Figure 12b. It can be seen that with the increase in Gr content, the degree to which the samples return to the initial state gradually increases. After 10 min, the stress in the sample is gradually released and the sample is gradually stretched, as shown in Figure 12c. Finally, the sample is close to the initial flat state for 1 h later. From the experimental results, it can be seen that the rubber-based composite prepared in this paper has good flexibility.

## 4. Conclusions

In conclusion, graphene/chopped glass fiber filled rubber-based composites were prepared by the two-roll milling and hot-pressing process. Due to the shearing force of the roller during the preparation process, both chopped glass fiber and graphene showed directional arrangement, which could be seen from the cross-sectional morphology of the material. With the increase in the contents of chopped glass fiber, the tensile strength of the rubber matrix composites increased first and decreased later. The tensile strength of the pure rubber in the T and W directions were 20 and 21 MPa. When the chopped glass fiber content was 1 wt.%, the tensile strength in the T and W directions both reached their maximum values, 27 and 28 MPa. Compared with pure rubber, the improvements were 35% and 33%, respectively. At the same time, the elongation at break of the material in both directions was maintained at 16%. The addition of graphene enhanced the flexibility of the material and improved the elongation at break of the material. When the content of glass fiber added was fixed at 1 wt.% and graphene was 1.5 wt.%, the elongation at break of the materials in both directions were 1.9- and 2.4-times than that of pure rubber. The material had good elongation at break and flexibility. Next, the dielectric properties of the material in the frequency of 2.6–18 GHz were measured, and the difference between the T and W directions of the X band was emphasized. The result showed that the directional arrangement of the additives led to the difference in the dielectric properties of the material in the two directions. At 8.2 GHz, when the addition of graphene was 2 wt.%, the real part of complex permittivity in the T direction was 4.8, while in W direction was 4.13. Calculating the input impedance of the material when the graphene content was 2 wt.% under different thicknesses, it was found that the impedance values in different directions were different, and the impedance performance of the material could be adjusted by using preparation method for the material.

## Data Availability

The authors declare that all data supporting the findings of this study are available within the article.
